# Concerning the Welfare of the Medical Society of the County of Erie1Presidental address delivered at the eighty-first annual meeting of the Medical Society of the County of Erie, held January 14, 1902.

**Published:** 1902-02

**Authors:** William C. Phelps

**Affiliations:** Buffalo, N.Y.


### Concerning the Welfare of the Medical Society of the County of Erie.[1]

By WILLIAM C. PHELPS, M.D., Buffalo, N.Y.

IT IS usual for a retiring president to bring to the attention of the society anything which especially pertains to its welfare. I have concluded that a few words concerning the society itself will be of possible benefit to it.

The Medical Society of the County of Erie is the oldest medical organisation in this city or county. According to data furnished me by the secretary, this society has existed since 1821, which was the next year after Erie County was set off from the County of Niagara. The Society, however, really has existed since 1808 for during that year Dr. Chapin perfected the organisation of the Niagara County Medical Society and the Erie County Medical Society was really its successor. Dr. Cyrenius Chapin was the first president, and he together with twenty-three others were the charter members. Erie County has been represented in the State Medical Society since the year 1823, and it has had among its members those who have become famous in the state and nation and throughout the world. I will not weary you with reciting the names of these eminent men, for they will recur to you all.

This society has been, during all its existence, a sort of clearing-house, if one may use the expression, for medical matters concerning the profession in this city and county. It has had conferred upon it important powers. For many years, through its board of censors, it had the power to issue licenses to those found competent to practise medicine, and these licenses were recognised throughout the state and throughout the United States. The board also has the power to bring proceedings in the courts against quacks of all degrees, and this feature of the powers of this society has never been more prominent than during the last few years under the able chairmanship of our distinguished ex-president, Dr. Coakley. It may also regulate the fees which its members may demand for their services, and its action is binding upon its members. On one occasion these fees were fixed not only for private practice but also for attendance in public institutions, and it is a matter of history that in this respect it overstepped its legal powers, for the courts, review-

1. Presidental address delivered at the eighty-first annual meeting of the Medical Society of the County of Erie, held January 14, 1902.

ing the action taken by the society in suspending some of its members, declared that it had no authority to enforce such a resolution in the manner in which it did.

It meets but twice a year and by reason of its legal rights has to transact a great amount of business not strictly scientific. Members resident in the city, or rather village, in 1831 organised the Medical Society of the Village of Buffalo. After ten years of successful work it took the name of the Buffalo Medical Association, and continued until it became absorbed by the Academy of Medicine. The fact that it was found necessary at so early a date, to hold meetings for strictly scientific work, may give us a hint as to whether it is not wise for the Medical Society of the County of Erie to hold sessions during which no business shall be transacted. We have a very large membership in the towns who have very little opportunity of attending meetings where scientific subjects are presented and discussed. They do not belong to the Academy of Medicine, nor to the medical clubs of the city. The Medical Society of the State of New York has found it necessary or advisable to hold a session for purely scientific purposes, and I would suggest that this society consider the propriety of holding meetings quarterly instead of twice a year; business to be transacted at the first and third, and medical subjects only at the second and fourth meetings or sessions.

An objection may be urged here that medical societies are already more numerous in the city than is profitable for those who are connected with them, and this prompts me to say a few words concerning the cause of the formation of some of them as it concerns this society.

At a very early date in the history of the American Medical Association, a code of medical ethics was adopted and made binding upon all its members. This code was written, as I have been informed, by Isaac Hayes, who for many years was the editor of the *American Journal of the Medical Sciences*. His character was so high, his ability so great, and his sense of honor so well known that it was thought best to commission him to draw up a code rather than have it prepared by a committee of the society. He did his work so well that it was unanimously adopted and was for many years respected and obeyed without question.

I think there is no doubt that had conditions remained unchanged it would still have the same obedience and respect rendered it as at the time of its adoption. Among its provisions was one that members in regular standing should not consult with practitioners of medicine who held to an exclusive theory of practice. That was meant to include, among others, homeopaths and eclectics. At that time these two classes of practitioners were not required to possess the knowledge of the science and art of medicine, which members of the regular profession considered absolutely necessary for one to possess in order to safely care for those needing the services of the physician and surgeon. In the case of the homeopathic persuasion little, if any, attention was paid to anything more than their materia medica and the same was true of the eclectic. Very little time and study were required to become worthy, in the estimation of these two classes of practitioners, to become their associates. They were densely and dangerously ignorant. As an illustration of this point I will relate a circumstance personal to myself.

In the town from which I came, as a medical student to Buffalo, lived an eclectic physician who acquired great wealth and reputation as a physician. His wife met me and I told her that I was going to Buffalo to study medicine. She asked me to what school I was going and how long would it take me. I told her that I was intending to attend the Buffalo Medical College and that it would be three years at least before I would receive my diploma. She told me that I was making a great mistake; that if I would come over to their house she would interest the doctor in my case, and that she knew he would allow me to use his books and study in the office, and that in six weeks I would be ready to begin the practice of medicine! She said that was all the time that the doctor had ever studied and that he had never required any of his students to spend any longer time.

In a few years a great change took place among these two classes of physicians as to the desirability and necessity of medical education. With commendable zeal they set themselves to work to remedy what they knew was a great evil among them. Schools were established, and preliminary education was required. Satisfactory examinations before the granting of diplomas were insisted upon, and as a result they soon had schools which taught all the branches of medicine probably as well as they were taught in the schools of the regular profession, excepting, of course, their specialties, materia medica and therapeutics. This improved condition of medical education on their part soon brought an educated class of students, fully equal to those attending the regular medical schools, and as they graduated and went out into practice, they became recognised as educated professional gentlemen. They could not be called quacks any longer. Treating them as quacks did not injure them, but injured those rather of the regular profession who adopted that plan of treatment toward them. In common with all applicants for license to practise they now must pass a final examination before the state licensing board.

About twenty years ago the Medical Society of the State of New York, after a warm discussion, by a majority vote declared that it would no longer be bound by the code of ethics of the American Medical Association. As I understand the matter, the chief objection was to that provision which prohibited members of the regular profession from meeting in consultation with those who practised an exclusive theory of medicine, meaning homeopaths and eclectics. But the society became, as I think, too radical and instead of stopping at absolving themselves from their allegiance to this one provision of it, threw the whole code over. I think that a code of procedure, similar if not exactly the same as that which has been in force so many years is necessary, with the exception of the clause to which the State Medical Society objected.

It teaches physicians what their duties are to each other; to their patients and to the public; the duties of the public to the profession; the duties of the patient to the physician, and thus contains what the medical man needs for his every-day work.

Recently a young medical man came to my office for advice as to what was the proper course to pursue in a consultation matter with two other physicians. He said that he had never heard anything about how he should act under those circumstances, for the reason that he had never been in a physician's office as a student. He had been a student of the university. The matter of being a student nowadays amounts to almost nothing as far as being a pupil of the alleged preceptor is concerned. In early times students were closely associated with their preceptors and they spent all their time in the office, excepting when they were attending medical lectures. They thus learned the ethics of the profession. But at the present time the majority of students are in the same condition of ignorance as to medical ethics as was the young physician who called upon me. I told him it was a very simple proposition and informed him where he would learn all about it,—that he would find it in the code of ethics of the American Medical Association. He said that he had never heard of it and he was glad to know that there were printed rules which would give him the information he desired. He said he had asked several young physicians of his acquaintance what to do and they were as ignorant as he. He could not understand why the medical schools which he had attended had not given some instructions on these points.

A code, then, I believe, is necessary and as I said before, I think it was a mistake that it was bodily rejected by the state medical society; but when it comes to the question of being prohibited from meeting a reputable, legally authorised medical man in the treatment of a case, I, for one, object and am of the opinion that the state medical society did right. I believe that the matter should be left entirely to the physician himself, as it is a matter that concerns him more than any one else. If he feels disposed or thinks it to be his duty to assist a reputable, legally authorised practitioner of medicine in any case, I believe that he has a right to do so, and, I do not believe that it is the concern of any other physician.

On this simple proposition the Medical Society of the State of New York was split in twain. The minority members formed a new organisation and called it the State Medical Association. From that association district branches have been formed, the fourth district of which includes the city of Buffalo and meets here. In addition, county medical associations have recently been formed, so that the Erie County Medical Society instead of being the sole representative of a state organisation finds itself with two rivals, the District Branch and the County Association affiliated with the State Medical Association. This condition of things can not but result in our injury. The secretary tells me that at present on our books we have enrolled 471 members. The members of the state medical association organisations in this vicinity either are or have been members of our society. Some have resigned, and if a spirit of intolerance, which has begun to show itself, should continue and become more radical all members of the state medical association, who are members of the Erie county medical society, will be obliged to resign.

I base this opinion on the fact that a member of our society, who was also a member of the state medical association and who is a member of the staff of the Erie county hospital wrote to Dr. Ferguson, of the state medical association, stating that on the staff of the county hospital were four homeopathic practitioners, that he was liable at any time to be called to see patients under their care in consultation, and asking for instructions. The reply which he received was that he must resign from the county hospital on account of the homeopathic practitioners on the staff with him, or he must resign his membership in the State Medical Association. He resigned his membership. There are forty more of us connected with the Erie county hospital who are liable at any moment to receive the same summons and, carried to its logical conclusion, I cannot see how a member of the county society can be allowed to be a member of the county medical association. Should this be carried out on the lines I have indicated our society must, of course, lose a large number of members and it will be prevented from receiving as members, a large number who otherwise would join us, so that it will not be long before new practitioners in Buffalo will be solicited, as is done at a caucus, to join either the county society or the county association. The code or no code question will not influence them as they know little and care less about it.

The revenues of our society have fallen off considerably in the last four years. The secretary tells me that he and the treasurer have been formerly paid for their services, but on account of the embarrassed condition of the treasury they will be willing to continue their services without pay. We will be unable to continue the prosecution of the army of quacks which will surely come to Buffalo, when it is learned that there is little danger of being disturbed in their nefarious work. Growing out of this condition of things there will be opportunities for misunderstandings and outright quarrels. Cliques will be formed and a united profession which once clustered around the old Erie county medical society will be a thing of the past. Looking at it in a still larger light, if no settlement of the difficulties is reached, it is not at all improbable that a new National Society will be formed and then we will have the spectacle, not only of warring county and state medical organisations, but also of national associations.

I am told that the formation of a National Medical Society has been more than talked of. The medical profession of the United States has been urging upon the National Government the desirability, the propriety, and the necessity of a medical officer having a seat in the Cabinet. What a spectacle the profession would make of itself, were two National organisations to appear at the Capitol to urge such a measure! How ridiculous the whole thing is when we stop for a moment to think what the cause of it all is. Simply whether a medical man shall not have the right to see a sick person, associated with a legally authorised physician if he and the physician and the patient and the patient's friends so desire? That is all there is of it and there is only one way in which the matter can ever be settled and that must be the way of a compromise.

Having observed the matter from the beginning, I am certain that the state medical society will never readopt that portion of the code to which it objected. I am also certain that the National Medical Association will never admit delegates from the state medical society while that society stands on record as having repudiated its whole code of medical ethics.

If the two warring factions were nations and had armies they would go out on the tented field and fight it out, and the one defeated would give up what they had contended for. That is not possible in this case. Wise men must get control of both organisations. At present, I am informed there is little hope that any compromise can be effected.

"Whom the gods would destroy they first make mad," and that is the condition in which these two factions are at present, but it does seem to me that in the near future a compromise may be arranged and that as a result of it, the profession in Buffalo will be saved from being split up into factions. The spectacle of two rival state organisations in existence will disappear and the Erie County Medical Society will once more resume its place as the central representative body of the profession of the County of Erie.

146 Allen Street.

